# Endothelin A (ETA) and Endothelin B (ETB) Receptor Subtypes Potentiate Epidermal Growth Factor (EGF)-Mediated Proliferation in Human Asthmatic Bronchial Airway Smooth Muscle

**DOI:** 10.7759/cureus.28333

**Published:** 2022-08-24

**Authors:** Mohannad A Almikhlafi, Koorosh Haghayeghi, Alice Gardner

**Affiliations:** 1 Pharmacology and Toxicology, Taibah University, Madinah, SAU; 2 Pharmaceutical Science, Massachusetts College of Pharmacy and Health Sciences, Worcester, USA

**Keywords:** endothelin, epidermal growth factors, airway remodeling, cell proliferation, broncheal asthma

## Abstract

Background

Asthma is a chronic disease characterized by chronic inflammation, reversible airway obstruction, airway hyperresponsiveness (AHR), and airway remodeling. One of the important features of asthma is airway remodeling, which plays a central role in airflow limitation. Airway remodeling involves numerous changes in the bronchial walls, including airway smooth muscle (ASM) cell hypertrophy and hyperplasia. Studies have shown that ASM hyperplasia in asthma is mediated by the increased production of mitogens. Endothelin-1 (ET-1) has been shown to induce proliferation and function as a co-mitogen in vascular and ASM. In patients with asthma, plasma and bronchoalveolar lavage fluid have been shown to have elevated ET-1 levels, which have been linked to airway remodeling and airflow obstruction in severe asthma. This study investigates the role of ET-1 in proliferation, the receptor subtype mediating its effect, and the signaling pathway.

Methodology

Normal and asthmatic bronchial airway smooth muscle (BASM) cells were seeded into 5 × 10^3 ^cells/well. Cell proliferation was assayed using 5-bromo-2’-deoxyuridine (BrdU) incorporation. Confluent cells were treated with different concentrations of ET-1 in the presence or absence of the epidermal growth factor (EGF). Signaling pathways were explored using pretreatment of BASM with antagonists 15 minutes before ET-1/EGF stimulation.

Results

In asthmatic BASM, ET-1 (0.1 nM) functions as a co-mitogen in the presence of EGF (10 nM), showing a significantly greater effect on asthmatic BASM proliferation compared with normal BASM. The ETA receptor antagonist BQ-123 (10-1,000 nM) significantly reduced the proliferative effect of ET-1/EGF on asthmatic BASM more than normal BASM. Moreover, the effect of ETB antagonist BQ-788 (1,000 nM) or pretreatment with the ETB agonist S6C (1-10 nM) followed by co-treatment with EGF in asthmatic BASM showed a small but significant decrease when pretreated with the inhibitor and increased with the agonist, thereby suggesting that the co-mitogenic effect of ET-1 is mainly via the activation of ETA receptors, with a small contribution by the ETB_ _receptors in asthmatic BASM. Finally, pertussis toxin (PTX) pretreatment (25 and 50 ng/mL) showed that EGF and ET-1/EGF mitogenic and co-mitogenic signaling utilizes Gi/0-mediated transactivation by EGF and ET receptors, especially in asthmatic BASM, leading to the activation of Ras-ERK-PI3K pathways. Enhanced ERK and PI3K effects on proliferation suggested that these kinases modulate the co-mitogenic effect of ET-1 in asthmatic BASM. Enhanced cross-talk between ET and EGF receptors may be a potential mechanism contributing to airway remodeling in asthmatic BASM.

Conclusions

ET-1 enhances the mitogenic effect of EGF predominantly via the ETA receptor in asthmatic BASM with the activation of Ras, ERK, and PI3K. The cross-talk mechanism between ET and EGF receptors may be a potential therapeutic target to prevent the progression of airway remodeling in ASM in patients with asthma.

## Introduction

Airway remodeling is an important aspect of lung diseases. Asthma is a chronic, inflammatory disease of the lungs, characterized by airway narrowing, airway hyperresponsiveness (AHR), presence of active inflammatory cells and inflammatory mediators, and structural changes in the airway [1]. Structural changes, also known as airway remodeling, are one of the important features of asthma and were first described in 1922 by Hubert and Koessler in cases of fatal asthma. Airway remodeling involves structural changes to the airways such as loss of epithelial integrity, thickening of the basement membrane, subepithelial fibrosis, goblet cells and submucosal gland enlargement, increased smooth muscle mass, decrease in cartilage integrity, and increased airway vascularity [2-4]. Of these changes, remodeling studies suggest that the increase in airway smooth muscle (ASM) proliferation is the most important factor that contributes to the increased responsiveness of the asthmatic airway.

Increased ASM number is a pathological characteristic that contributes signiﬁcantly to airﬂow limitation and AHR in asthma [5]. ASM proliferates in response to two distinct types of receptors: (1) receptor tyrosine kinases (RTKs) and (2) G protein-coupled receptor (GPCR) [6,7]. RTKs are activated by polypeptide growth factors, e.g., platelet-derived growth factor (PDGF), epidermal growth factor (EGF), basic fibroblast growth factor-2 (FGF-2), and insulin-like growth factor, while GPCRs are activated by contractile agonists, e.g., serotonin, thromboxane, endothelin-1 (ET-1), and leukotriene D4 [8,9].

Airway remodeling is mediated by different mitogens, including ET, which has been implicated in the pathogenesis of several diseases, including bronchial asthma [10]. Patients with different types of asthma have been shown to have an increase in the level of ET-1, both in plasma [11] and bronchoalveolar lavage fluid [12], and the level of ET-1 corresponds with the severity of the disease. Also, the ET-1 gene (*EDN1*) expression is elevated significantly in patients with different subtypes of asthma compared with healthy subjects [13]. Moreover, inhalation of nebulized ET-1 leads to reduced forced expiratory volume in one second (FEV1) in asthmatic patients [13]. On the other hand, it has been shown to regulate proliferation in rabbits and normal human ASM [10]. ETs, including all three subtypes, have been shown to produce their effects through the activation of ET receptors. ET receptors belong to the superfamily of GPCR and mediate their effects via ETA and ETB receptors [14]. Several studies have observed the effect of ET-1 in vascular and normal ASM proliferation [15,16] and the co-mitogenic role that potentiates the proliferative effect of other growth factors such as EGF and PDGF [17,18].

Most of the asthma studies have been carried out in animal tracheal smooth muscle, while in this study, human ASM has been used. Furthermore, many studies have investigated the effect of ET-1 in normal smooth muscle, while in this study, it will be carried out in both normal and diseased ASM cells. Finally, in this study, the contribution of ETA and ETB receptors to the proliferative effects of ET and the signaling pathways will be determined in both asthmatic and non-asthmatic bronchial airway smooth muscle (BASM) cells.

This article was previously presented as a meeting abstract at the American Thoracic Society 2014 international conference on May 16, 2014.

## Materials and methods

Cell culture

Human BASM cells were purchased (Lonza, Walkersville, MD, USA), fed every other day with Smooth Muscle Cell Basal Medium (SmBM) supplemented with Smooth Muscle Growth Medium-2 (SmGM-2) SingleQuots: 5% fetal bovine serum (FBS), 0.1% insulin, 0.2% human FGF-2 (hFGF-2), 0.1% gentamicin, 0.1% human EGF (hEGF; Lonza). Cells were grown in T25 flask TPP® (MIDSCI, St. Louis, MO, USA) at 5 × 10^3^ cm^-2^ and subcultured according to the manufacturer’s protocol.

Cell proliferation

When 85%-90% confluent, BASM was then seeded in a 96-well TPP® (MIDSCI) at 5 × 10^3^ cells/well for 24 hours in SmBM supplemented with SmGM-2 singleQuots and serum starved for 48 hours in SmBM with 0.1% BSA. Then the cells were treated with ET-1 (Anaspec, Fremont, CA, USA) for 48 hours alone or in combination with EGF (10 nM; Enzo Life Sciences, Farmingdale, NY, USA). Inhibitors or appropriate vehicles were added to the cells 15 minutes before ET-1/EGF treatment. Cell proliferation was carried out using the 5-bromo-2’-deoxyuridine (BrdU) Cell Proliferation Assay Kit (Cell Signaling Technology, Danvers, MA, USA). BrdU solution (10×), which detects BrdU incorporation, was added to all the samples for 24 hours and aspirated thereafter. Following the manufacturer’s protocol, cells were fixed onto a 96-well plate using the manufacturer’s solution and incubated at room temperature for 30 minutes. Following the removal of the fixing solution, a detection antibody solution (1×) was added for 1 hour at room temperature, followed by appropriate washing three times with wash buffer (1× phosphate-buffered saline [PBS]). After that, horseradish peroxidase (HRP)-conjugated secondary antibody solution (1×) was added for 30 minutes to each well, followed by appropriate washing three times with wash buffer (1× PBS). TMB substrate was added to each well for 30 minutes. Stop solution (sulfuric acid) was added to prevent further color change. The samples were read in a multimode microplate reader (Synergy H1BioTek, Winooski, VT, USA) at 450 nm.

Statistics 

Data were analyzed by analysis of variance (ANOVA) using GraphPad Prism versions 6.00 for Windows (GraphPad Software, San Diego, CA, USA). Results were presented as mean ± SEM, *n *= 4. *P* < 0.05 will be considered statistically significant.

## Results

Effect of ET-1 on normal and asthmatic BASM proliferation

**Figure 1:**
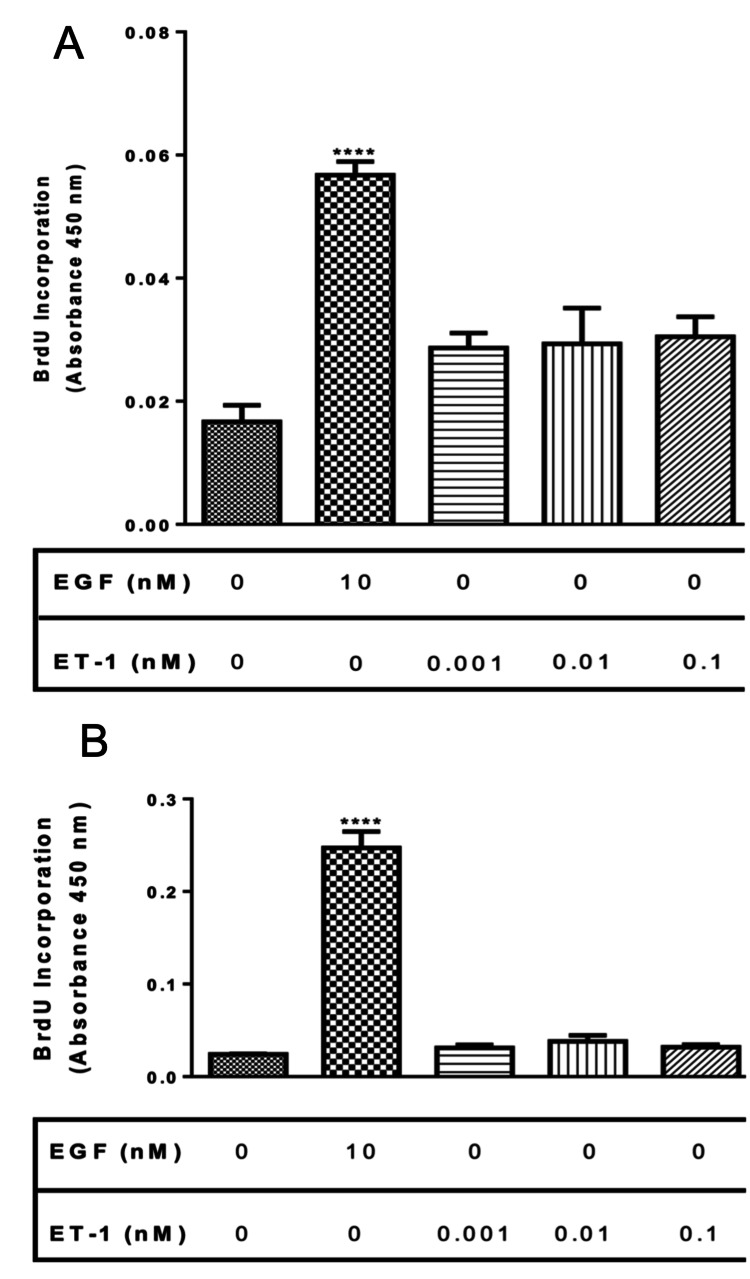
Effect of ET-1 on normal and asthmatic BASM proliferation (A) Normal BASM and (B) asthmatic BASM were grown to confluence and then growth-arrested for 24 hours in serum-free media with 0.1% BSA. Next, they were treated with EGF (10 nM), a positive control, ET-1 (0.001, 0.01, and 0.1 nM). As described in the Materials and Methods section, BrdU incorporation was measured after 18 hours of stimulation. DNA synthesis was compared with that obtained from cells treated with serum-free media (control). Data represent mean ± SEM from six to eight separate experiments. *Significance vs. control. ****P < 0.001. BASM, bronchial airway smooth muscle; BrdU, 5-bromo-2’-deoxyuridine; BSA, bovine serum albumin; EGF, epidermal growth factor; ET-1, endothelin-1.

Human normal and asthmatic BASM were used to examine the effect of ET-1 on BASM cells proliferation using BrdU incorporation, as described previously in the Materials and Methods section. Normal and asthmatic BASM were treated with EGF (10 nM) or different concentrations of ET-1 (0.001-0.1 nM). Results showed that normal BASM treated with EGF showed 259% increase in proliferation, while asthmatic BASM showed 934% increase in proliferation. On the other hand, normal BASM treated with different concentrations of ET-1 exhibited 89.7% (0.001 nM), 75% (0.01 nM), and 111% (0.1 nM) increase in proliferation compared with control (Figure 1, Panel A). In addition, asthmatic BASM treated with ET-1 showed 38.54% (0.001 nM), 79% (0.01 nM), and 43% (0.1 nM) increase in proliferation compared with control (Figure 1, Panel B).

Effect of ET-1 on EGF-induced proliferation on BASM

**Figure 2:**
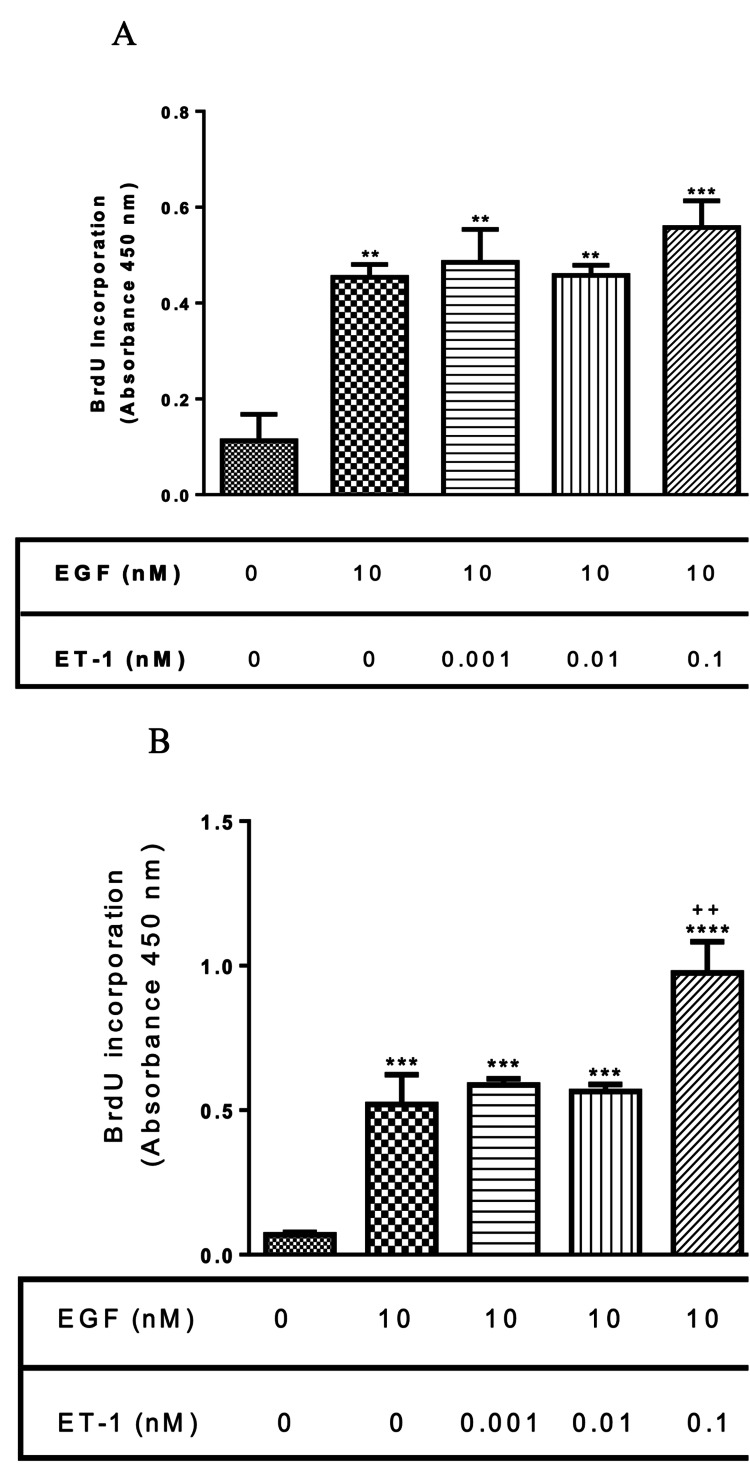
Effect of ET-1 and EGF co-treatment on normal and asthmatic BASM proliferation (A) Normal BASM and (B) asthmatic BASM were grown to confluence and then growth-arrested for 24 hours in serum-free media with 0.1% BSA, followed by treatment with EGF (10 nM) alone or co-treated with ET-1 (0.001, 0.01, and 0.1 nM). As described in the Materials and Methods section, BrdU incorporation was measured after 18 hours of stimulation. Data represent mean ± SEM from six to eight separate experiments. *Significance vs. control; ^+^significance vs. EGF; **P < 0.01; ***P < 0.001; ****P < 0.001; ^++^*P* < 0.01. BASM, bronchial airway smooth muscle; BrdU, 5-bromo-2’-deoxyuridine; BSA, bovine serum albumin; EGF, epidermal growth factor; ET-1, endothelin-1.

To examine the effect that ET-1 has on EGF-induced proliferation, normal and asthmatic BASM were treated with EGF (10 nM) alone or in combination with different concentrations of ET-1 (0.001-0.1 nM). The co-treatment of EGF with ET-1 did not show any significant change in proliferation compared with EGF-treated BASM (Figure 2, Panel A). On the contrary, asthmatic BASM treated with EGF and 0.1 nM ET-1 significantly increased proliferation by 120% compared with EGF-treated BASM (Figure 2, Panel B).

Effect of ETA and ETB receptors subtype on ET-1-induced enhancement of proliferation

**Figure 3:**
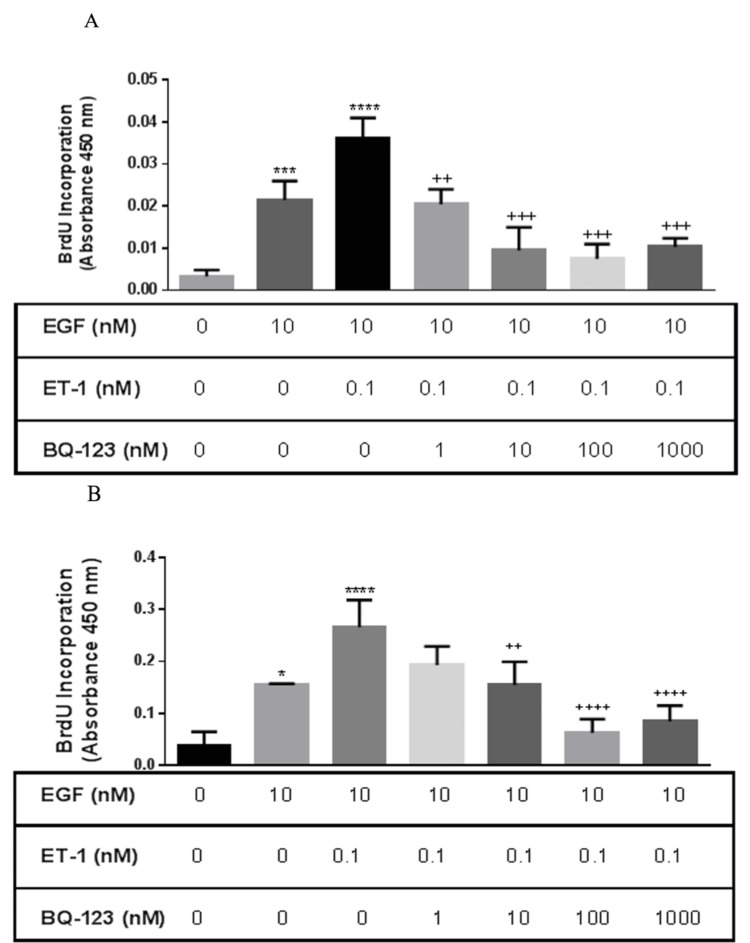
Effect of the ETA receptor antagonist on asthmatic and normal BASM proliferation in response to EGF and ET-1 (A) Normal BASM and (B) asthmatic BASM were grown to confluence and then growth-arrested for 24 hours in serum-free media with 0.1% BSA. Cells were pretreated with BQ-123 (1, 10, 100, and 1,000 nM), a selective ETA receptor antagonist for 30 minutes, followed by co-treatment with EGF (10 nM) and ET-1 (0.1 nM). As described in the Materials and Methods section, BrdU incorporation was measured after 18 hours of stimulation. Data represent mean ± SEM from four to six separate experiments. *Significance vs. control; ^+^significance vs. ET-1/EGF. ***P < 0.001; ****P < 0.001; ^++^*P *< 0.01; ^+++^*P *< 0.001; ^++++^*P *< 0.001. BASM, bronchial airway smooth muscle; BrdU, 5-bromo-2’-deoxyuridine; BSA, bovine serum albumin; EGF, epidermal growth factor; ET-1, endothelin-1; ETA, endothelin A receptor.

In order to identify the receptor subtype responsible for ET-1 effect on EGF proliferation, normal BASM cells were pretreated with the ETA receptor antagonist (BQ-123) and ETB receptor antagonist (BQ-788). Normal BASM pretreated with BQ-123 (1, 10, 100, and 1,000 nM) significantly decreased EGF/ET-1-induced proliferation: 42%, 71.82%, 77%, and 71.46%, respectively, compared with the EGF/ET-1-treated group (Figure 3, Panel A). 

**Figure 4:**
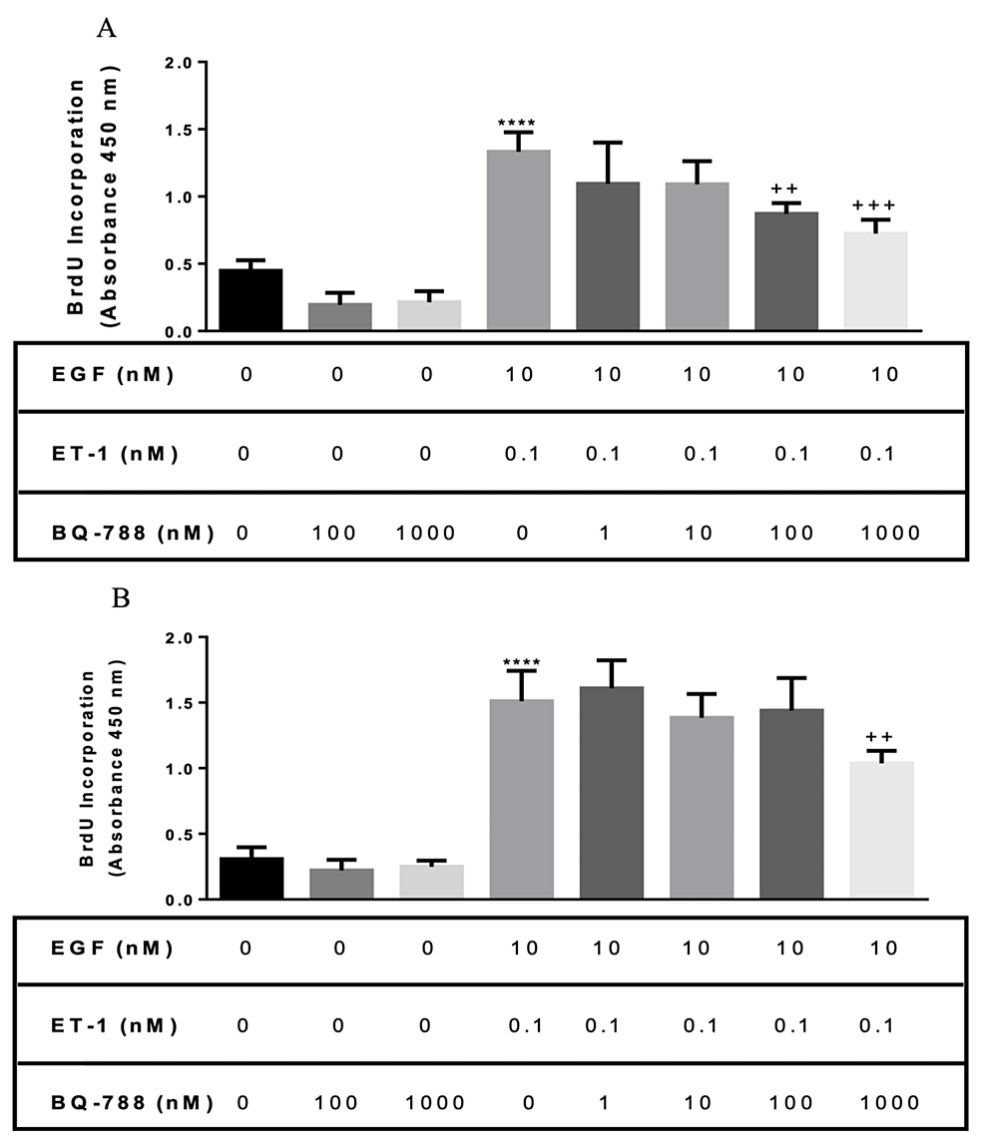
Effect of the ETB receptor antagonist on asthmatic and normal BASM proliferation in response to EGF and ET-1 (A) Normal BASM and (B) asthmatic BASM were grown to confluence and then growth-arrested for 24 hours in serum-free media with 0.1% BSA. Cells were pretreated with BQ-788 (1, 10, 100, and 1,000 nM), a selective ETB receptor antagonist for 30 minutes, followed by co-treatment with EGF (10 nM) and ET-1 (0.1 nM). As described in the Materials and Methods section, BrdU incorporation was measured after 18 hours of stimulation. Data represent mean ± SEM from four to six separate experiments. *Significance vs. control; ^+^significance vs. ET-1/EGF; ****P < 0.001; ^++^*P *< 0.01; ^+++^*P *< 0.001. BASM, bronchial airway smooth muscle; BrdU, 5-bromo-2’-deoxyuridine; BSA, bovine serum albumin; EGF, epidermal growth factor; ET-1, endothelin-1; ETB, endothelin B.

In contrast, ETB receptor antagonist (BQ-788) pretreatment had no effect at low concentration. However, a high concentration of BQ-788 (100 and 1,000 nM) was able to significantly decrease EGF/ET-1-induced proliferation (Figure 4, Panel A).

Similarly, asthmatic BASM were pretreated with BQ-123 and BQ-788. Results showed that BQ-123 (10, 100, and 1,000 nM) significantly decreased the mitogenic effect of ET-1 on EGF: 26.26%, 47%, 72.69%, and 66.71%, respectively, compared with the EGF/ET-1-treated group (Figure 3, Panel B). On the other hand, only 1,000 nM of BQ-788 was able to significantly decrease proliferation compared with the EGF/ET-1-treated group (Figure 4, Panel B). These results suggest that ET-1 could augment the mitogenic effect of EGF in BASM mainly via the ETA receptor. 

Effect of the ETB receptor agonist on proliferation

**Figure 5:**
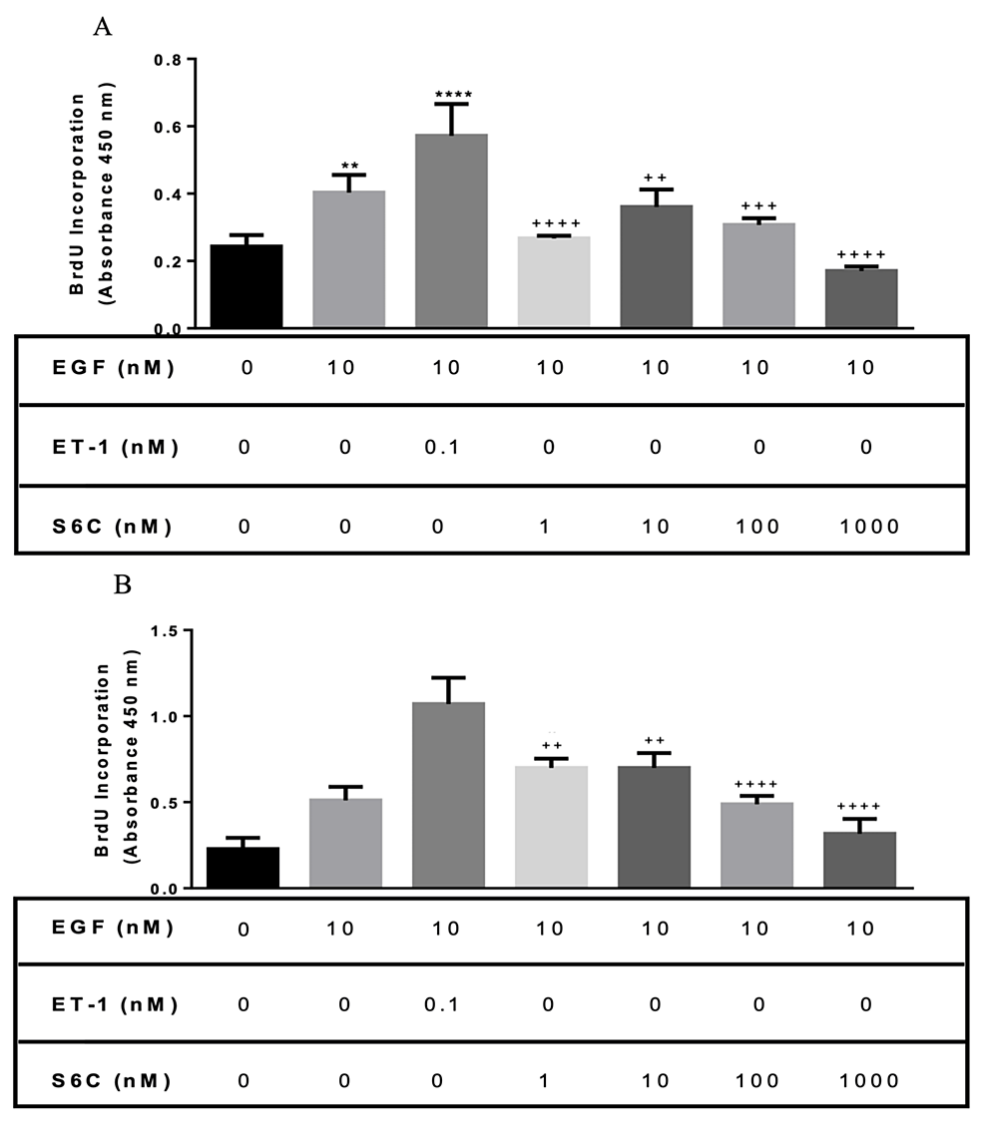
Effect of the ETB receptor agonist on asthmatic and normal BASM proliferation in response to EGF and ET-1 (A) Normal BASM and (B) asthmatic BASM were grown to confluence and then growth-arrested for 24 hours in serum-free media with 0.1% BSA. Cells were co-treated with EGF (10 nM) and S6C (1, 10, 100, and 1,000 nM), a selective ETB receptor agonist for 48 hours. As described in the Materials and Methods section, BrdU incorporation was measured after 18 hours of stimulation. DNA synthesis was compared with that obtained from cells treated with 0.1% BSA in a serum-free media (control). Data represent mean ± SEM from four to six separate experiments. *Significance vs. control; ^+^significance vs. EGF/ET-1; **P < 0.01; ****P < 0.001; ^++^*P *< 0.01; ^+++^*P *< 0.001; ^++++^*P *< 0.001. BASM, bronchial airway smooth muscle; BrdU, 5-bromo-2’-deoxyuridine; BSA, bovine serum albumin; EGF, epidermal growth factor; ET-1, endothelin-1; ETB, endothelin B; S6C, Sarafatoxin 6C.

To confirm that the ETB receptor has a minor effect on EGF-induced proliferation, a selective ETB receptor agonist Sarafatoxin 6C (S6C) was used to treat BASM in combination with EGF. In normal BASM, different concentrations of S6C (1, 10, 100, and 1,000 nM) co-treated with EGF showed a significantly lower proliferation than EGF/ET-1: 53.21%, 36.93%, 46.26%, and 70%, respectively (Figure 5, Panel A). Similarly, in asthmatic BASM, EGF co-treated with S6C (1-1,000 nM) showed a significantly lower proliferation than the EGF/ET-1-treated group (Figure 5, Panel B).

Stimulation of ET receptors induces proliferation via Gi/0 in normal human ASM

**Figure 6:**
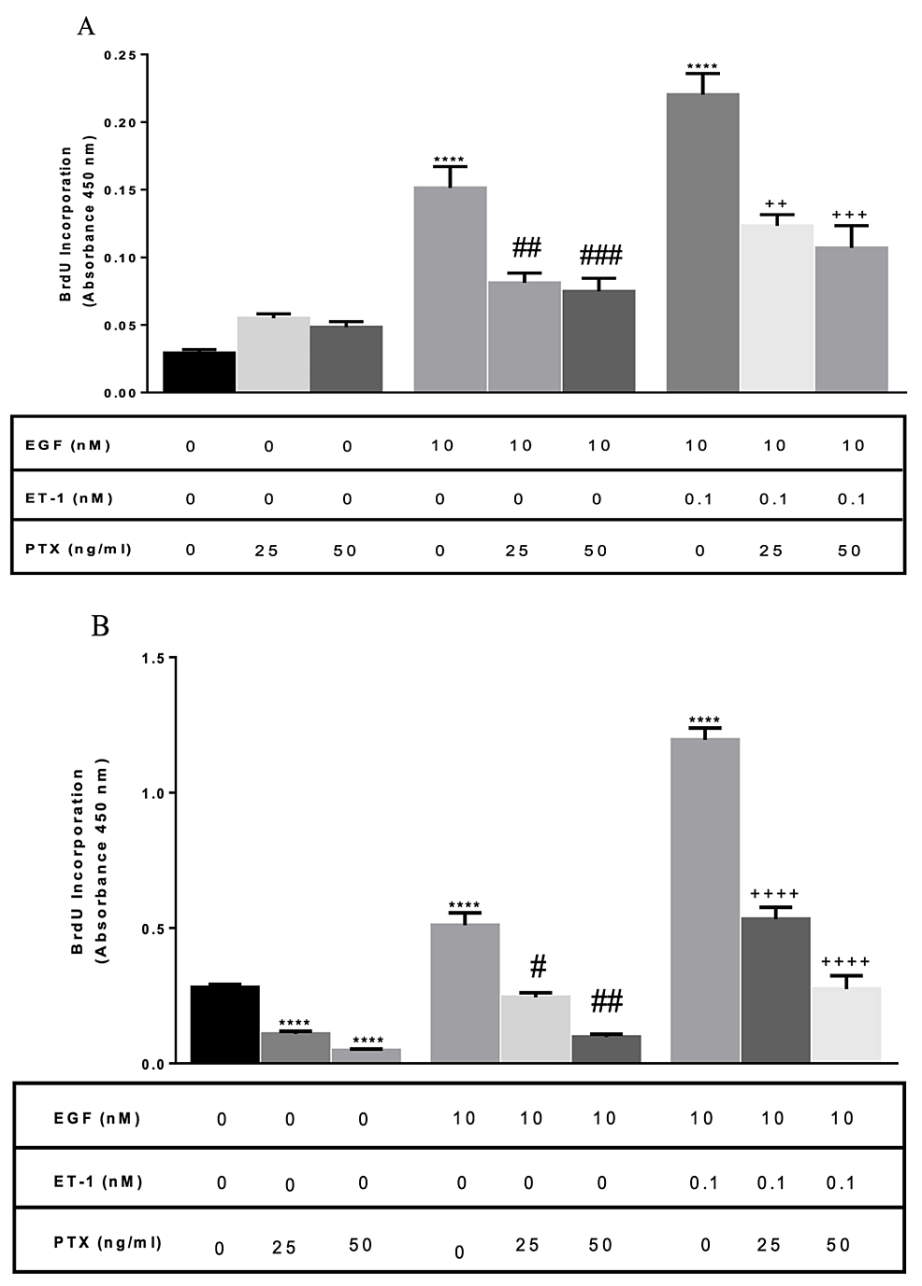
Role of Gi/0 in normal and asthmatic BASM (A) Normal BASM and (B) asthmatic BASM were grown to confluence and then growth-arrested for 24 hours in serum-free media with 0.1% BSA. Cells were pretreated with PTX (25 and 50 ng/mL), a Gi inhibitor, overnight. Then the cells were treated with EGF (10 nM) and EGF/ET-1 (0.1 nM). As described in the Materials and Methods section, BrdU incorporation was measured after 18 hours of stimulation. Data represent mean ± SEM from four to six separate experiments. *Significance vs. control; ^#^significance vs. EGF; ^+^significance vs. ET-1/EGF; ****P < 0.001; ^++^*P *< 0.01; ^+++^*P *< 0.001; ^++++^*P *< 0.001; ^#^*P *< 0.05; ^##^*P *< 0.01; ^###^*P *< 0.001. BASM, bronchial airway smooth muscle; BrdU, 5-bromo-2’-deoxyuridine; BSA, bovine serum albumin; EGF, epidermal growth factor; ET-1, endothelin-1; Gi, G protein-coupled receptor type i; PTX, pertussis toxin.

In order to determine the G protein family subtype responsible for proliferation induced by ET-1, BASM cells were pretreated with pertussis toxin (PTX) 25 and 50 ng/mL. PTX is known to catalyze the ADP-ribosylation of the α subunits that prevent Gi/0 protein from coupling with the receptor. Normal BASM pretreated with PTX (25 and 50 ng/mL) decreased EGF-induced proliferation: 46% and 45%, respectively, compared with the EGF alone treated group. In addition, pretreatment of cells with PTX (25 and 50 ng/mL) decreased EGF/ET-1-induced proliferation: 38%, and 46%, respectively, compared with EGF/ET-1-treated group (Figure 6, Panel A). 

Similarly, asthmatic BASM pretreated with PTX (25 and 50 ng/mL) decreased EGF proliferation: 55% and 82%, respectively, compared with the EGF-treated group. Also, pretreatment of cells with PTX (25 and 50 ng/mL) decreased EGF/ET-1-induced proliferation: 55% and 76%, respectively, compared with the EGF/ET-1 treated group (Figure 6, Panel B). These results indicate that the ETA receptor is linked to the Gi/0 receptor subtype of G protein-coupled receptors. In addition, there is a transactivation between the EGF receptor and G protein-coupled receptors on the surface of BASM.

Proliferation signaling pathway

**Figure 7:**
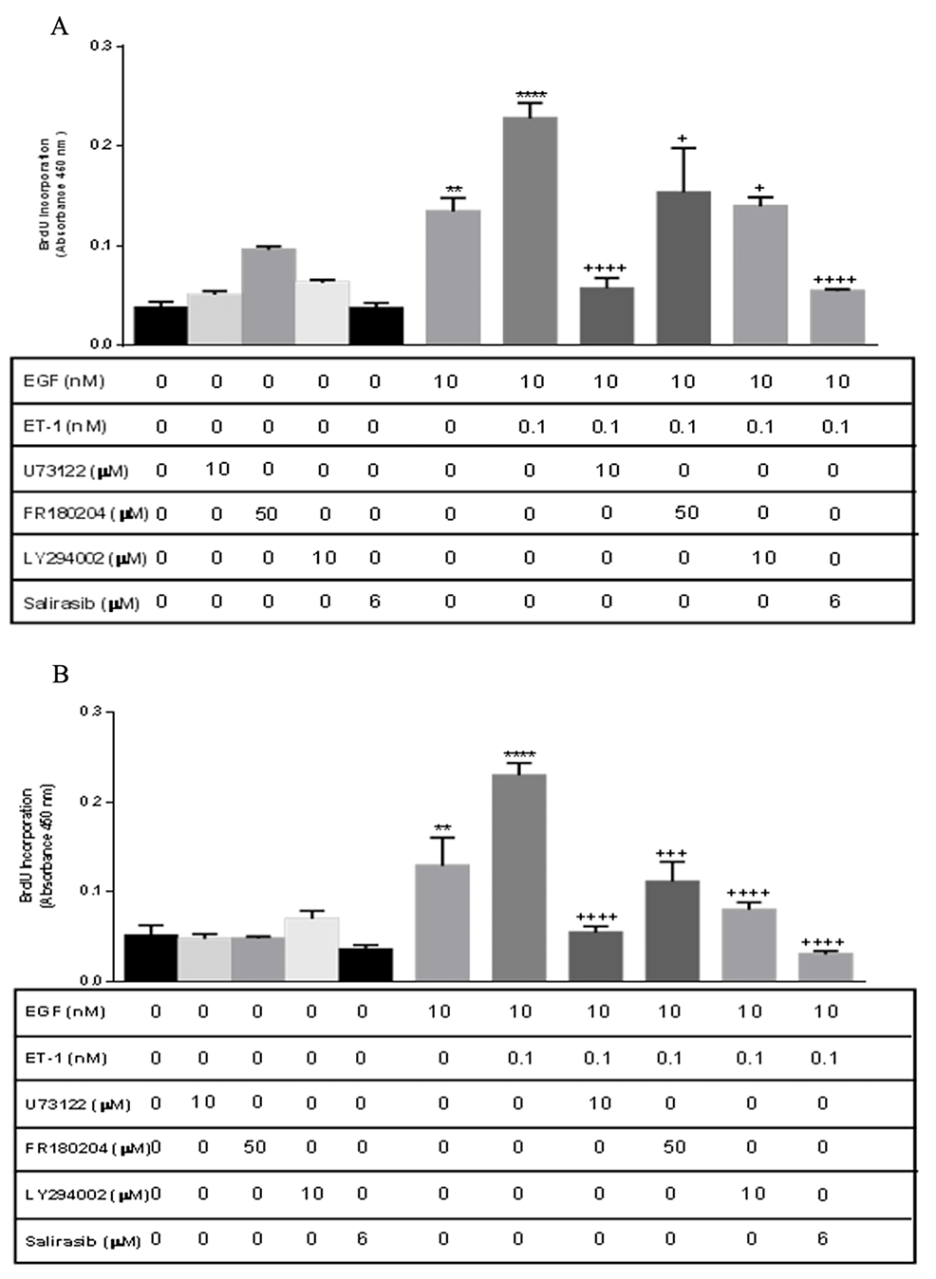
ET/EGF proliferation signaling pathway in BASM (A) Normal BASM and (B) asthmatic BASM were grown to confluence and then growth-arrested for 24 hours in serum-free media with 0.1% BSA. Cells were pretreated with U73122 (10 μM), a PLC inhibitor; FR180204 (50 μM), an ERK inhibitor; Salirasib (6 μM), a Ras inhibitor; and LY294002 (10 μM), a PI3K inhibitor for 30 minutes. Then cells were treated with EGF (10 nM) and EGF/ET-1 (10 nM/0.1 nM). As described in the Materials and Methods section, BrdU incorporation was measured after 18 hours of stimulation. Data represent mean ± SEM from four to six separate experiments. *Significance vs. control; ^+^significance vs. ET-1/EGF; **P < 0.01; ****P < 0.001; ^+^*P *< 0.05; ^+++^*P *< 0.001; ^++++^*P *< 0.001. BASM, bronchial airway smooth muscle; BrdU, 5-bromo-2’-deoxyuridine; BSA, bovine serum albumin; ERK, extracellular signal-regulated kinases; EGF, epidermal growth factor; ET-1, endothelin-1; PLC, phospholipase C; PI3K, phosphoinositide 3-kinase.

To investigate the signaling pathway of EGF/ET-1 proliferation, BASM cells were pretreated with extracellular signal-regulated kinase (ERK), phosphoinositide 3-kinase (PI3K), Ras, and phospholipase C (PLC) inhibitors. Normal and asthmatic BASM were pretreated with U73122 (10 μM), a PLC inhibitor; FR180204 (50 μM), an ERK inhibitor; Salirasib (6 μM), a Ras inhibitor; and LY294002 (10 μM), a PI3K inhibitor, in the presence or absence of EGF/ET-1. In normal BASM, U73122, FR180204, LY294002, and Salirasib significantly decreased EGF/ET-1 proliferation: 70.07%, 32.6%, 34.4%, and 77.32%, respectively, compared with the EGF/ET-1 treated group (Figure 7, Panel A). Similarly, asthmatic BASM pretreated with U73122, FR180204, LY294002, and Salirasib significantly decreased EGF/ET-1 proliferation: 71.69%, 44.37%, 57.31%, and 85.67%, respectively, compared with the EGF/ET-1-treated group (Figure 7, Panel B).

**Figure 8:**
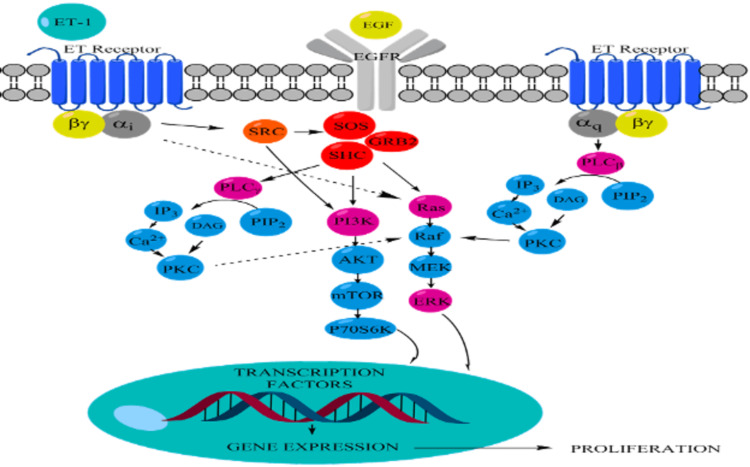
Proposed signaling pathway for ET-1/EGF-induced proliferation EGF, epidermal growth factor; ET-1, endothelin-1.

These results indicate that not only ET receptors mediate their effect through Gi/0 but also Gq is involved in the signaling pathway. Downstream signaling exhibited the involvement of ERK, Ras, and PI3K in normal and asthmatic BASM proliferation (Figure 8).

## Discussion

In this study, the following five major findings have been demonstrated: (1) ET-1 alone has small but significant effects on the proliferation of normal and asthmatic BASM, but it is not significant; (2) ET-1 significantly potentiates the mitogenic effects of EGF in asthmatic BASM; (3) the effects of ET-1 is mainly mediated by the ETA receptor in both normal and diseased BASM, while the ETB receptor has a minimal effect on asthmatic BASM; (4) ET-1 potentiates the co-mitogenic effect through the activation of a Gi/0 and Gq receptor subtype; and (5) the co-mitogenic effect of ET-1 is mediated by activation of Ras, ERK, and PI3K signaling pathways. 

These results suggest that ET-1 can augment the mitogenic effects of growth factors in BASM via the activation of ETA receptors, which may play a major role in mediating human ASM remodeling in patients with asthma. The data demonstrate that ET-1 alone has a minimal effect on proliferation, which is consistent with what was previously seen in tracheal smooth muscle cells [17-19]. ET-1 is found to be able to potentiate proliferation in PDGF- and EGF-treated tracheal ASM [17,18]. EGF alone has a mitogenic effect that significantly increases proliferation in normal and asthmatic BASM compared with control. However, the combination of ET-1 and EGF increases the proliferation of EGF asthmatic BASM but not in normal cells, which suggests that ET-1 is a potent co-mitogen in asthmatic BASM. 

The biological effects of the ET family, especially ET-1, are mediated via the activation of G protein-coupled receptors. A selective ETA antagonist (BQ-123) and ETB antagonist (BQ-788) are used to determine the ET receptor subtypes responsible for the mitogenic influencing the effect of ET-1. The potentiation effect of ET-1 on EGF is significantly reduced by an ETA antagonist, BQ-123, and modestly by the ETB antagonist, BQ-788, in both normal and diseased BASM. The role of ETA receptor in ET-1-induced proliferation is also found in rat tracheal smooth muscle [18], human tracheal smooth muscle [17], and human pulmonary artery [20]. Furthermore, ETB agonist (S6C) does have a potentiation effect on EGF in asthmatic BASM but is absent in normal BASM. These data would suggest that the co-mitogenic effect of ET-1 is mainly via the activation of ETA receptors, with a small contribution by the ETB receptors in asthmatic BASM. 

The downstream signaling of ET-1-induced proliferation has not been fully elucidated. The data presented in this study suggest that the augmentation of BASM cells growth may occur due to the activation of common downstream signaling molecules linked to GPCR and RTK. In the present series of experiments, a Gi/0 inhibitor (PTX), a PLC inhibitor (U73122), a PI3K inhibitor (LY294002), a Ras inhibitor (Salirasib), and an ERK inhibitor (FR180204) are used to investigate the signaling pathway that regulates ET-1-induced potentiation and proliferation of BASM in the presence of EGF. These data suggest that ETA receptor-mediated DNA synthesis is regulated by multiple subtypes of GPCR. The role of Gi/0 was demonstrated using the pretreatment with PTX, which was able to decrease the proliferation of EGF alone. The concept of the RTK-GPCR complex was previously introduced; however, this study confirmed that there is transactivation between the two receptors in normal and asthmatic BASM [21]. In addition, PTX treatment can significantly decrease the effect of ET-1/EGF-induced mitogenesis in normal and asthmatic cells, similar to its effect on tracheal ASM from guinea pigs [22]. The potential role of Gq was tested using a PLC inhibitor, which significantly decreases ET-1/EGF-induced proliferation, similar to its effect on ET-1/PDGF in rat tracheal ASM [18]. This study also shows the involvement of PI3K, as demonstrated by a previous study that used EGF to treat ASM with the PI3K inhibitor (LY294002) [23]. Using the PI3K inhibitor, LY294002, caused a greater reduction in the mitogenic effect of co-treatment in asthmatic BASM compared with normal BASM.

Activation of Ras has been known to be important in growth factor signaling and regulation of proliferation. The role of Ras in regulating cell growth and proliferation has been proven by many research studies, for example, when bovine placental trophoblast cells were treated with EFG, a high expression of Ras was observed, which led to an increase in proliferation [24]. On the other hand, ET-1-induced proliferation through activation of Ras was demonstrated in astrocytoma cells [25]. The data presented in this study confirm the role of Ras in proliferation. When the Ras inhibitor, Salirasib, is used, it induces a significant reduction in proliferation. These data are similar to a previously published study that showed the ability of Salirasib to decrease EGF-mediated proliferation in gliosarcoma cells [26]. 

The role of ERK in proliferation is tested using the ERK inhibitor. ERK is known to be one of the key molecules responsible for cell proliferation in various cell lines through activation of cyclin D, which leads to cell cycle progression. In rat tracheal smooth muscle cells, DNA synthesis is attenuated upon inhibition of ERK [27], and activation of ERK is observed following ET-1 treatment [18]. In this study, it is found that ERK plays a role in ET-1/EGF-mediated proliferation. Inhibition of ERK1/2 using FR180204 can reduce proliferation induced by the co-treatment of ET-1 and EGF to a greater extent in asthmatic BASM compared with normal BASM. The data presented in this study are consistent with previous studies that demonstrated the role of ERK1/2 activation in mediating DNA synthesis induced by ET-1.

## Conclusions

Airway remodeling is a key feature in asthma. ET-1 plays a major role in airway remodeling in patients with bronchial asthma. ET-1 alone cannot increase proliferation. However, in the presence of EGF, ET-1 has a co-mitogenic effect that increases EGF proliferation greater than EGF alone in asthmatic BASM. A proposed signaling pathway for ET-1/EGF-induced proliferation in asthmatic BASM suggests that ET-1 binds to two subtypes of ET receptors: ETA and ETB. The co-mitogenic effect of ET-1 is predominantly via ETA, while ETB has a minimal effect on proliferation. In addition, the ET-1 effect is mediated by the activation of the Gi/0 and Gq receptor subtypes of GPCR, which subsequently activate Ras, PLC, ERK, and PI3K signaling pathways. Enhanced ERK and PI3K effects on proliferation suggest these kinases modulate the co-mitogenic effect of ET-1 in asthmatic BASM. Targeting ET receptors and the downstream signaling pathway could be a potential target to inhibit airway remodeling in asthmatic patients.
